# The SGK3/GSK3β/β-catenin signaling promotes breast cancer stemness and confers resistance to alpelisib therapy

**DOI:** 10.7150/ijbs.104850

**Published:** 2025-03-19

**Authors:** Tingting Kang, Yuanfang Wang, Yaxin Jiang, Shunjie Chen, Na Lin, Minyan Guo, Haotu Zhu, Di Tang, Xiaofan Ding, Mian He

**Affiliations:** 1 Scientific Research Center, The Seventh Affiliated Hospital of Sun Yat-sen University, Shenzhen, China.; 2 Department of General Surgery, The Seventh Affiliated Hospital of Sun Yat-sen University, Shenzhen, China.; 3 Faculty of Health Sciences, University of Macau, Macau SAR, China.; 4 Department of Oncology, The Seventh Affiliated Hospital of Sun Yat-Sen University, Shenzhen, China.; 5 MOE Frontiers Science Center for Precision Oncology, University of Macau, Macau SAR, China.; 6 Guangdong Provincial Key Laboratory of Digestive Cancer Research, The Seventh Affiliated Hospital of Sun Yat-sen University, Shenzhen, China.

**Keywords:** Alpelisib, drug resistance, stem cell, SGK3, β-catenin

## Abstract

Drug resistance is the leading cause of death in patients with advanced tumors. Alpelisib, a selective PIK3CA inhibitor, has been recently approved for treating advanced breast cancer. However, drug resistance is inevitable, and the mechanisms behind alpelisib-associated resistance remain elusive. To address this problem, we established an alpelisib-resistant breast cancer cell model and confirmed that the resistant cells exhibited enhanced abilities in colony formation, migration, anti-apoptosis, spheroidization, tumor formation and metastasis. Further analysis revealed that *SGK3* was significantly upregulated in alpelisib-resistant cells, which was strongly associated with tumor stemness. Additionally, we observed that SGK3 promoted tumor cell stemness by activating GSK3β/β-catenin signaling pathway, leading to the resistance to alpelisib in breast cancer. Finally, we demonstrated that SGK3 inhibitor could restore the sensitivity of resistant cells to alpelisib. Collectively, these findings suggest that SGK3 could be a novel therapeutic target for breast cancer patients who developed resistance to alpelisib.

## Introduction

Breast cancer is the most common cancer among women worldwide and the second leading cause of cancer-related deaths in women 1. In recent years, the incidence of breast cancer has been steadily increasing. Primary treatments for breast cancer include surgical resection, endocrine therapy, chemoradiotherapy, targeted therapy and immunotherapy. Despite recent advances in treatments and disease managements, about 20% of patients can develop drug resistance and metastases, resulting in a 5-year survival rate of less than 30% 2.

Endocrine therapy, including estrogen antagonist tamoxifen and aromatase inhibitors, is the mainstay of treatment for patients with hormone receptor-positive (HR+) breast cancer 3. However, more than 30% of patients with early HR-positive breast cancer relapse within 15 years after receiving the treatment, and about 20% patients treated with aromatase inhibitors relapse within 9 years 4. It has been reported that such resistance to endocrine therapy was mainly due to the abnormal activation of the PI3K/AKT signaling pathway 5, among which *PIK3CA* mutations are found in approximately 40% of patients, causing constitutive activation of the α isoform (p110α) of PI3K and the subsequent regulation of cell growth, proliferation, metastasis, angiogenesis in tumor cells 5. Therefore, inhibitors targeting PI3K/AKT signaling pathway, *e.g.*, alpelisib, inavolisib, ipatasertib, MK-2206 and sirolimus, could significantly prolong the survival of patients with HR+ advanced breast cancer 5-7. Among these, alpelisib serves a PI3K inhibitor by selectively blocking p110α and has been approved by the Food and Drug Administration (FDA) to treat advanced or metastatic breast cancer following progression on or after an endocrine-based regimen in 2019. Recent clinical trials also demonstrated that *PIK3CA*-mutated cancers were more sensitive to alpelisib 7, displaying a significant better progression-free time 8. Despite alpelisib was able to generate promising results in these trails, resistance to alpelisib post a long-time administration is still inevitable. Therefore, there is an urgent need to further uncover the actionable molecular mechanisms underlying resistance to alpelisib.

Cancer stem cells (CSCs) are characterized by the capacity in self-renewal, differentiation and continuous proliferation, resulting in metastasis, recurrence and drug resistance of breast cancer 9. Breast cancer stem cell (BCSC) was first identified and isolated by Al-Hajj from a patient-derived xenograft (PDX) model in 2003 10. BCSCs can be identified by several defined surface markers, including CD133, CD44, ALDH1, GD2 and Integrins 11. The major signaling pathways regulating BCSC include Wnt, Notch, Hedgehog, PI3K/Akt/mTOR, and HER2 12. Therapies targeting stem cell-associated genes and pathways, e.g. Wnt/β-catenin pathway, have been shown to be effective in eliminating CSCs 9, 13. However, it remains unclear whether BCSCs contribute to alpelisib resistance.

Serum and glucocorticoid-regulated kinase (SGK) mainly includes three isoforms: *SGK1*, *SGK2* and *SGK3* 14. The SGK catalytic domain shares 50% homology with AKT and both can promote cancer cell proliferation 15-17. Studies have found that *SGK3* was transcriptionally activated by estrogen receptor ESR1 and high SGK3 expression was associated with poor prognosis of HR+ breast cancer patients 18. In breast cancer cells with *PIK3CA* mutation, PI3K inhibition resulted in enhanced estrogen receptor function 19, 20, which might subsequently upregulate the expression of SGK3. Moreover, SGK3 was activated in a dose-dependent manner following the treatment of a PI3K inhibitor and promoted liver cancer stem cell expansion 21, 22. Recent studies have also emphasized the role of SGK3 in drug resistance, including resistance to AKT inhibition, mTORC1 inhibitors and endocrine therapy 19, 23, 24. However, the relationship between SKG3 and alpelisib-related resistance, as well as its role in BCSCs, remained elusive.

To address this problem, we have developed drug resistance toward alpelisib in two *PIK3CA*-mutant cell lines, *i.e.*, MCF7 and T47D and observed enhanced tumorigenicity and metastasis by both *in vitro* and *in vivo* experiments. Mechanistically, SGK3, a serine/threonine-protein kinase, compensated the loss of PI3K/AKT signal and re-activated downstream targets, enhancing the stemness of malignant cells and further leading to the resistance toward alpelisib. Fortunately, SGK3 inhibition could restore the sensitivity of resistant cells to alpelisib.

## Materials and Methods

### Cell culture and reagents

Both MCF7 and T47D breast cancer cells were kindly provided by Stem Cell Bank, Chinese Academy of Sciences. MCF7 cells were maintained in Dulbecco's modified Eagle medium (DMEM) and T47D cells were cultured in RPMI-1640 medium. Both DMEM and RPMI-1640 medium was supplemented with 10% FBS, 0.01mg/ml bovine insulin, 2mM L-glutamine and 100U/ml penicillin and streptomycin. Cells were incubated in an incubator at 37°C in humidified air with 5% CO2. Alpelisib (BYL719) (Selleck, S2814), inavolisib (MCE, HY-101562), tamoxifen (MCE, HY-13757A), PROTAC SGK3 degrader-1 (SGK3-IN) (AbMole, M10382) and VPS34 inhibitor 1 (VPS34-IN1) (MCE, HY-12795) were diluted in DMSO.

### Establishment of Alpelisib-resistant cells and stable cell lines

To build up the alpelisib-resistant cell model, MCF7 and T47D cells were incubated with alpelisib-containing medium. The initial alpelisib exposure concentration was 0.8μM. When cell growth was stable under this concentration, alpelisib concentration was gradually increased until the final concentration reached 10μM. The alpelisib-resistant cell model was confirmed by comparing the apelisib IC50 values of parental and resistant cells. Short tandem repeats (STR) profiling was performed to determine the cell line authentication.

To generate SGK3 overexpression or knockdown stable cells, plenti-SGK3 or PLKO.1-shSGK3 plasmids were transduced into breast cancer cell lines by lentivirus, respectively. Puromycin was added as a selection marker for stable cell lines. Western blot and qPCR were performed to validate the SGK3 expression in above stable cells.

### Cell viability assay and combinational index calculation

Cells (2,000/well) were seeded in 96-well plate in triplicates overnight and treated with drugs. Three days later, fresh medium containing 10% CCK-8 reagent was added and incubated at 37°C for 2 hours. Then the absorbance was detected at A=490nm by a microplate spectrophotometer. To calculate combinational index, the resistant cells were seeded and treated with escalating doses of BYL719 and SGK3-IN, cells viability was determined by CCK8 assay and combinational index was calculated by the CalcuSyn software. Experiments were repeated at least twice with three technical replicates.

### Flow cytometry

Parental or alpelisib-resistant cells were digested into single cells by EDTA solution and resuspend in PBS. To detect the proportion of early and late apoptotic cells, all floating and adherent cells were collected after serum starvation for 24 hours and then stained with FITC-Annexin V and PI using FITC-Annexin V/PI apoptosis kit (UElandy, F6002M) according to the manufacturer's protocol. To detect the stem cell population, breast cancer cells were stained with FITC-CD44 (Biolegend, 338803) and PE-CD24 (Biolegend, 311105) antibodies for 15 min on ice. Flow cytometry analysis was carried out on BD flow cytometer and data was analyzed by Flowjo software. Experiments were repeated at least twice with three technical replicates.

### Colony formation assay

For colony formation assay, 1,000 cells were seeded in 6-well plates and treated with drugs for 21 days. At the end of experiment, the colonies were fixed with 4% paraformaldehyde and stained with 0.1% crystal violet for colonies counting. Experiments were repeated at least twice with three technical replicates.

### 3D sphere formation assay

For tumor sphere formation assay, 5,000 cells were plated in 24-well ultra-low adhesion plate and grown in serum-free DMEM-F12 supplemented with 20 μg/L bFGF, 10 μg/L EGF and 2% B27. Three weeks later, spheres were counted by an inverted microscope and the number of tumor spheres were calculated in each group. Experiments were repeated at least twice with three technical replicates.

### Transwell migration assay

Parental and alpelisib-resistant cells were digested and resuspend in serum-free medium. Then, 50,000 cells were placed on the upper layer of a transwell cell culture insert with permeable membrane and fresh medium containing 10% FBS was placed below the cell permeable membrane. After 48 hours of incubation, the cells that had migrated through the membrane were fixed in methanol and stained by crystal violet. The number of migrated cells were scored for at least ten microscope fields. Experiments were repeated at least twice with three technical replicates.

### Immunofluorescence staining

Cells grown on glass coverslip were fixed with 4% paraformaldehyde and permeabilized by 0.5% Triton X-100 for 30 min. Then cells were blocked with 10% BSA solution and incubated with diluted SGK3 and β-catenin antibodies overnight at 4℃. After washed with PBS, cells were incubated with fluorescent-conjugated secondary antibodies for 1h at room temperature. Finally, cells were stained with DAPI and mounted with anti-fluorescent quench blocking solution. Images were captured by a confocal microscope. Experiments were repeated at least twice with three technical replicates.

### RNA extraction, cDNA synthesis and real-time PCR

The total RNA was extracted by FastPure Cell Total RNA Isolation Kit (Vazyme, RC112-01) according to manufacturer's instruction. First-strand complementary DNA was transcribed using HiScript III All-in-one RT SuperMix (Vazyme, R333-01). SYBR Green Premix Pro Taq HS qPCR kit (AG, AG11701) was used to performe the real-time polymerase chain reaction. The relative expression levels of target genes were calculated based on the ΔΔCT method where GAPDH was used as the housekeeping gene. The primer sequences used for real-time PCR were listed in Table S1. Experiments were repeated at least twice with three technical replicates.

### RNA data processing and pathway enrichment analysis

The raw RNA sequencing data was processed to filter the low-quality reads using Fastp (v0.23.4)^25^. The clean reads were then aligned to the human reference genome Hg38 using the STAR (v2.7.11a) 26. Quantification of gene expression levels were implemented by RSEM (v1.3.3) 27. The differentially expressed genes between alpelisib- resistant group and control group were identified by DeSeq2 (v1.40.2) 28. To gain insights into the pathways associated with SGK3, GO enrichment analysis for deregulated genes in SGK3 high and low luminal breast cancer patients was performed using the Gene Ontology resource by R package clusterProfiler (v4.6.2)^29^.

### Western blot analysis

RIPA lysis buffer containing 1×protease and phosphatase inhibitor cocktail (Beyotime, P1045) were used to extract protein from cells. Protein concentration was determined by Detergent Compatible Bradford Protein Assay Kit (Biosharp, BL1434A). The protein samples were resolved on SDS-PAGE and then transferred onto PVDF membranes. The membranes were blocked with 5% BSA for 1 hour at room temperature and incubated with diluted primary antibody overnight at 4°C with gentle shaking. The second day, the membranes were incubated with the secondary antibody at room temperature for 1 h. Finally, the bands were visualized by enhanced chemiluminescence. Primary antibodies used in this study were as follows: SGK3 (Santa Cruz, sc-166847), p-SGK3 (UpinBio, YP-Ab-10407), AKT (ABmart, T55561S), p-AKT (473) (ABmart, T40067F), S6 (Santa Cruz, sc-74459), p-S6 (235)(Santa Cruz, sc-293144), p-S6(240)(Santa Cruz, sc-293143), GSK3β (ABmart, T40069F), p-GSK3β (9)(ABmart, T40070F), β-catenin (ABmart, M24002F), p-β-catenin (543) (ABclonal, AP0579), p-β-catenin (675) (ABclonal, AP0795), CD44 (ABclonal, A19020), c-Myc (ABclonal, T55150), PARP (Santa Cruz, sc-8007), ESR1 (MCE, HY-P80663), E-Cadherin (Wanleibio, WL01482), N-Cadherin (Wanleibio, WL01047), Vimentin (ABmart, T55134), GAPDH (CST, 2118), Lamin A/C (CST, 13448). Experiments were repeated at least twice.

### *In vivo* animal studies

This study has used 6-weeks old female BABL/c nude mice which were purchased from Guangdong Medical Laboratory Animal Center. Mice were housed at laboratory animal center of Sun Yat-sen University and study protocols were approved by the Institutional Animal Care and Use Committee of Sun Yat-sen University. For intracardiac injection mice model, 100,000 parental or alpelisib resistant breast cancer cells stably expressing firefly luciferase were resuspended in 0.1 ml sterile PBS and injected into the left ventricle of nude mice (n=11 for each group) under anesthesia. From the second week, mice were injected intraperitoneally with D-fluorescein potassium salt every 3 days and *in vivo* imaging was performed to detect the metastasis of tumor cells in mice. The death of mice was recorded timely for further survival analysis. For xenograft mice model, a total of 5×10^6^ breast cancer cells were suspended in 0.1 mL PBS and orthotopically injected into the mouse mammary fat pads (n=5 for each group). When tumors reached an average size of 50 to 100 mm^3^, mice were randomized into 4 groups as following: a. shNC+PBS; b. shSGK3+PBS; c. shNC+BYL719 (50 mg/kg/day); d. shSGK3+BYL719 (50 mg/kg/day). Tumor growth was evaluated every 3 days and calculated by the following formula: Volume=length×width^2^/2. At the end of experiment, mice were killed and xenografts were fixed in 4% paraformaldehyde for further study.

### Statistical analysis

Statistical analysis was performed using GraphPad Prism 6 software and statistical significance was defined as *p* value less than 0.05. One-way ANOVA was used for multiple group comparison. Log-rank test was used for survival analysis. Comparison between two groups was conducted by use of unpaired two-sided Student`s *t* test.

## Results

### Establishment of alpelisib-resistant breast cancer cell model

**Figure 1:**
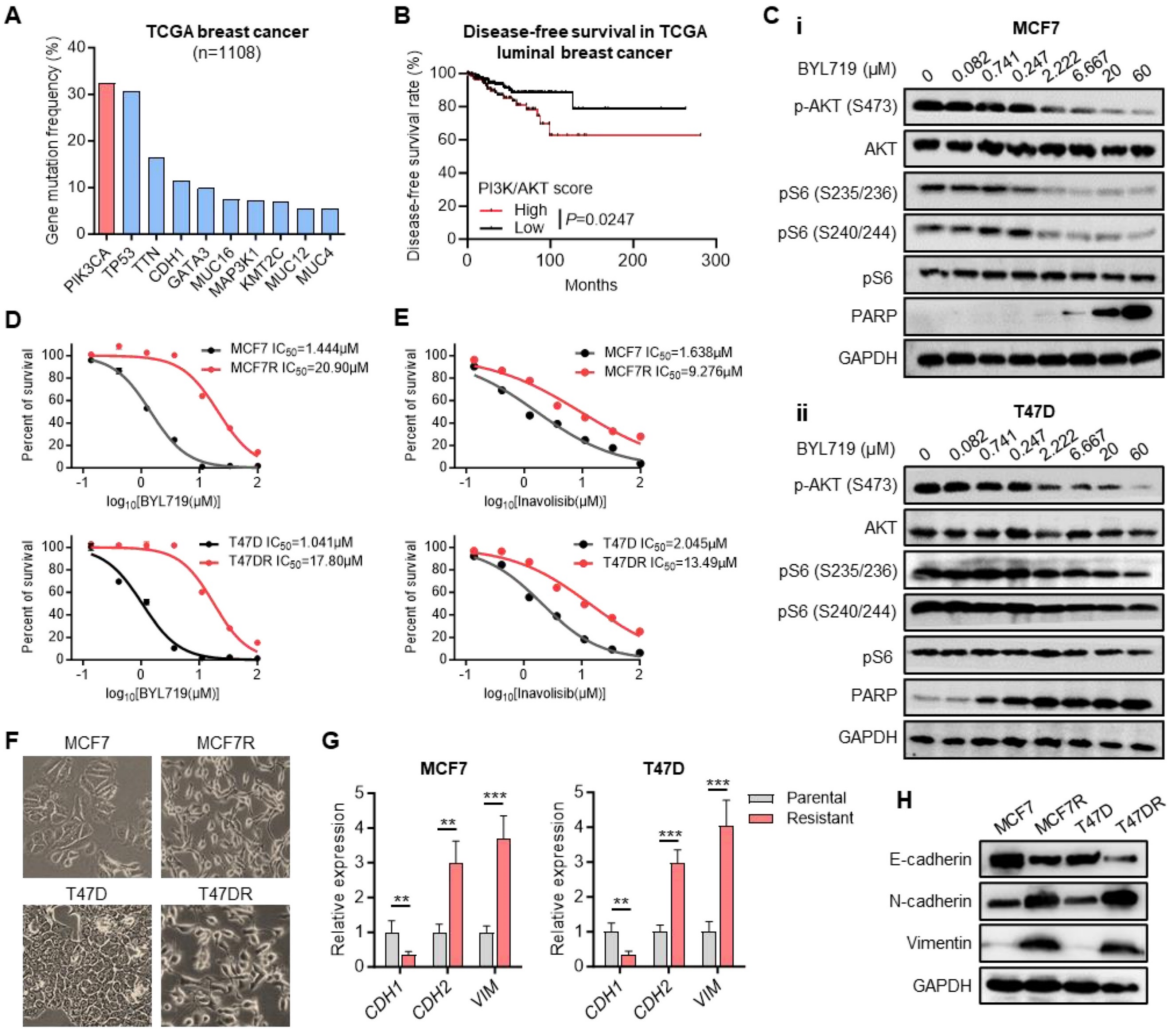
** Generation of alpelisib-resistant breast cancer cell lines.** (A) The most frequently mutated genes in breast cancer based on TCGA dataset. (B) Kaplan-Meier curves depicting luminal breast cancer patients with high and low PI3K/AKT signaling scores. (C) Western blot for total and phosphorylated AKT and S6 as well as apoptosis protein PARP in MCF7 and T47D cells treated with BYL719 for 4 hours. (D-E) Dose-response curves for BYL719 (D) and inavolisib (E) in parental and alpelisib-resistant breast cancer cell lines. (F) The morphology of parental cells and alpelisib-resistant cells. (G) The RNA expression levels of EMT markers in parental and alpelisib-resistant cells. (H) Western blot for EMT markers in parental and alpelisib-resistant cells. Unpaired *t* test, **p < 0.01, ***p < 0.001).

*PIK3CA* gene encodes the catalytic subunit alpha (p110α) of phosphatidylinositol 3-kinase (PI3K), which is frequently mutated in human cancers, especially in breast cancer 30. According to The Cancer Genome Atlas (TCGA) dataset, *PIK3CA* was the most frequently mutated gene in breast cancer occurring in more than 30% of cases, most of which were activating mutations (Figure 1A). Kaplan-Meier analysis indicated that the higher PI3K/AKT signaling scores could predict a shorter disease-free survival of luminal breast cancer patients (Figure 1B, Table S2). Alpelisib (BYL719), a selective inhibitor of p110α, has shown its synergistic antitumor effect with endocrine therapy in breast cancer patients harboring *PIK3CA* mutations 31, 32. Western blot results confirmed the inhibition of BYL719 on PI3K/AKT signaling pathway, showing the suppressed phosphorylation levels of AKT and S6 as well as elevated expression level of apoptotic marker PARP along with increased concentration of BYL719 in two *PIK3CA* mutated breast cancer cell lines MCF7 and T47D (Figure 1C).

To address the molecular mechanism of alpelisib-related drug resistance, alpelisib-resistant breast cancer cell models were established by adapting MCF7 and T47D cells to BYL719 from initial concentration 0.8μM to final 10μM. Next, the Cell Counting Kit-8 (CCK-8) assay was performed to confirm that the half maximal inhibitory concentration (IC50) of BYL719 in alpelisib-resistant cells MCF7R and T47DR had been increased more than ten-fold than those in parental cells (Figure 1D). Interestingly, these alpelisib-resistant cells also gained resistance toward another PI3Kα inhibitor, inavolisib (Figure 1E). Moreover, the morphology of MCF7 and T47D cells underwent a marked epithelial-mesenchymal transition (EMT) as resistance to alpelisib developed (Figure 1F). A marked decrease in E-cadherin and increase in N-cadherin and Vimentin was observed in alpelisib-resistant cell lines (Figure 1G-H), Therefore, we have successfully established the alpelisib-resistant breast cancer cell line model.

### The biological characteristics of alpelisib-resistant breast cancer cells

**Figure 2:**
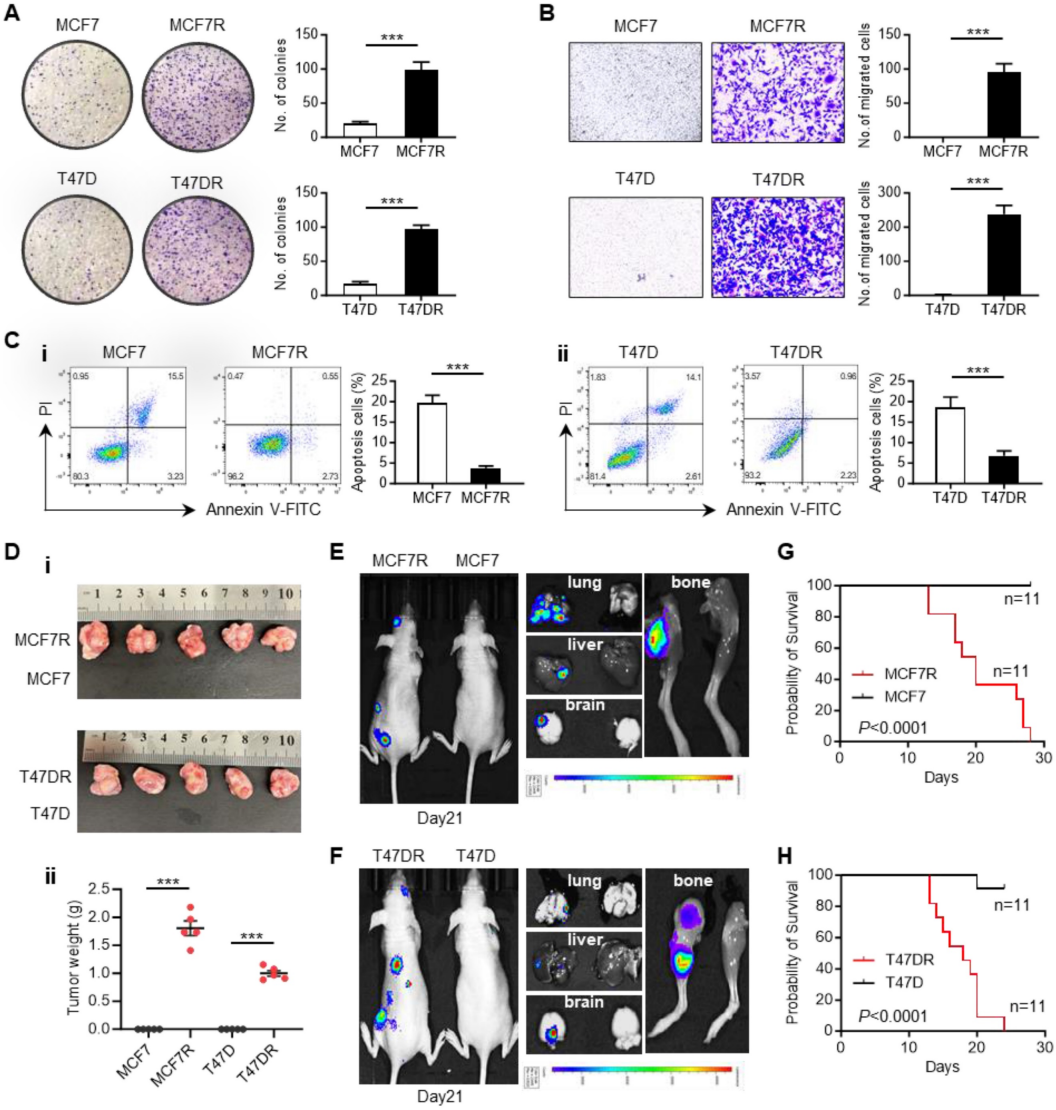
** The biological characteristics of alpelisib-resistant breast cancer cells.** (A) Colony forming abilities of parental cells and alpelisib-resistant cells. (B) Migratory abilities of parental cells and alpelisib-resistant cells. (C) The proportion of apoptotic cells in parental and alpelisib-resistant cells after 24 hr serum starvation detected by FITC-Annexin V/PI staining. (D) Representative images of tumor xenografts generated by orthotopically injection of parental and alpelisib-resistant cells (i) and tumor weights in each group (n=5) (ii). (E-F) *In vivo* bioluminescence images showing the metastasis of parental and alpelisib-resistant cell lines in the intracardiac injection mice model. (G-H) Kaplan-Meier curves depicting the overall survival of mice intracardially injected with parental and alpelisib-resistant cells (n=11). In (A-D): ***p < 0.001 by unpaired *t* test. In (G-H): p was calculated by log-rank test.

To explore the biological changes accompanying alpelisib resistance, a series of *in vitro* and *in vivo* experiments were conducted to characterize the differences between parental cells and alpelisib-resistant cells. Colony-formation assay showed that the alpelisib-resistant cells formed larger and more clones than the parental cells (Figure 2A). Transwell migration assay also confirmed that the migratory ability of alpelisib-resistant cells was significantly enhanced (Figure 2B). Annexin V/PI apoptosis assay was performed by showing the decreased proportion of apoptotic cells in the alpelisib-resistant cells after serum starvation, which demonstrated the enhanced anti-apoptotic ability in alpelisib-resistant cells (Figure 2C).

To assess the tumorigenicity of alpelisib-resistant cells, parental and alpelisib-resistant cells were orthotopically injected into the mouse mammary fat pad and only the resistant cells could form tumors without estrogen pellets supplementation, revealing the growth of alpelisib-resistant cells was estrogen-independent and their stemness property was also enhanced (Figure 2D and Figure S1A-B). Moreover, the intracardiac injection mice model was also applied to investigate the metastasis ability of alpelisib-resistant cells. *In vivo* bioluminescence imaging result showed that only the alpelisib-resistant cells could systemically metastasize to the brain, lung, liver, and femur of the mice within 1 month post-injection (Figure 2E-F), which consequently caused early death in mice intracardially injected with resistant cells (Figure 2G-H). Taken together, these results proved that the abilities of colony formation, migration, anti-apoptosis, tumorgenicity and metastasis were significantly increased in alpelisib-resistant cells.

### *SGK3* was upregulated in alpelisib-resistant breast cancer cells

**Figure 3:**
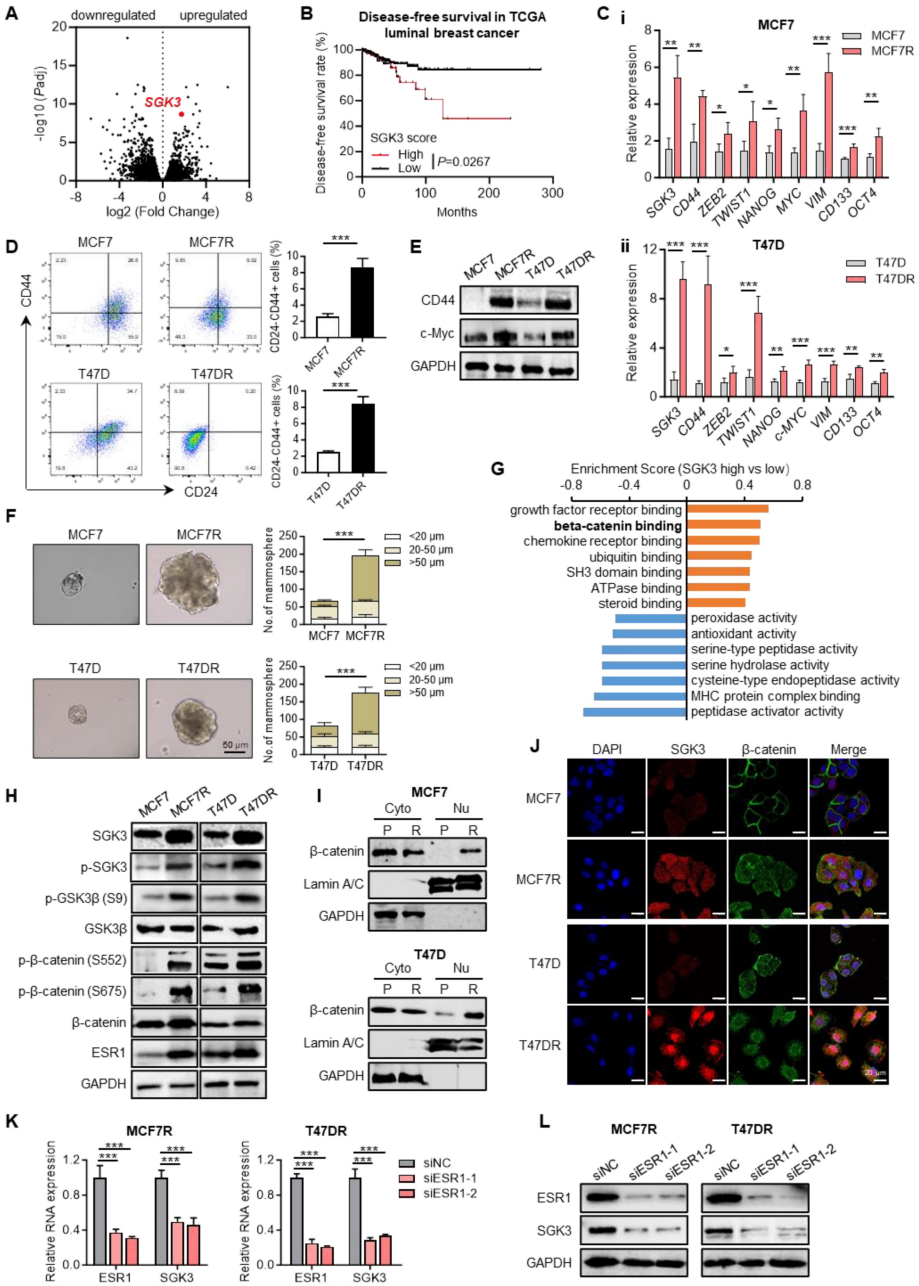
** SGK3 was significantly upregulated in alpelisib-resistant cell lines.** (A) RNA profiles showing the upregulation of *SGK3* in alpelisib-resistant cell lines. (B) Kaplan-Meier curves depicting luminal breast cancer patients with high and low SGK3 scores. (C) The RNA expression levels of *SGK3* and tumor stemness related markers in parental and alpelisib-resistant cells. (D) The proportion of CD24-/CD44+ breast stem cell population in parental and alpelisib-resistant cells. (E) Tumor spheres formation abilities of parental cells and alpelisib-resistant cells. (F) Western blot for CD44 and c-Myc protein levels. (G) GO enrichment analysis of differentiated expressed genes in luminal breast cancer patients with high and low SGK3 expression. (H) Western blot for total and phosphorylated SGK3, GSK3β, β-catenin and ESR1. (I) Western blot showing the protein levels of β-catenin in cytoplasm and nucleus. P: parental cell, R: resistant cells (J) Immunofluorescence staining for β-catenin localization. (K) The RNA expression levels of *ESR1* and *SGK3* after *ESR1* knockdown. (L) Western blots of ESR1 and SGK3 after knockdown of ESR1 in alpelisib-resistant cells. Unpaired *t* test, *p < 0.05, **p < 0.01, ***p < 0.001

To identify the key regulators that involved in alpelisib resistance, RNA profiles of parental and resistant cells were compared and *SGK3* was found to be significantly upregulated in MCF7R and T47DR cells, revealing that *SGK3* might play an important role in alpelisib resistance (Figure 3A).

Similarly, SGK3 expression was also markedly higher in luminal breast cancer patients who were resistant to a PI3K/mTOR inhibitor NVP-BEZ235 as estimated by the DeepDR analysis (Figure S2). In Kaplan-Meier analysis, high *SGK3* expression correlated with shorter disease-free survival of luminal breast cancer patients when compared with cases with low *SGK3* expression (Figure 3B). In addition, stemness related markers such as C*D44*, *CD133*, *MYC*, *OCT4*, *NANOG*, *TWIST1* and other genes were highly expressed in the resistant cells (Figure 3C). Previous studies have demonstrated that tumor cell stemness is closely related to drug resistance. Consequently, breast cancer stem cells were evaluated using flow cytometry, revealing that the proportion of CD24-/CD44+ cells was significantly higher in alpelisib-resistant cells compared to parental cells, as well as protein levels of CD44 and c-Myc (Figure 3D-E). Tumor spheroid formation assay also confirmed the tumor spheres formed by MCF7R and T47DR were significantly larger and more numerous than those formed by the parental cells (Figure 3F). Thus, these results demonstrated that the increased stemness phenotype was likely contributed to the acquired resistance toward alpelisib in breast cancer cells.

To find the potential downstream signaling pathways regulated by SGK3, RNA profiles of breast cancer patients with high and low SGK3 expression were compared. GO enrichment analysis suggested β-catenin binding was positively correlated with SGK3 (Figure 3G). As previously described, GSK3β/β-catenin was one of known downstream pathways of SGK3 and played a key role in mediating cancer stemness 21, then we check whether GSK3β/β-catenin pathway was activated in alpelisib-resistance cells. As expected, the phosphorylated GSK3β (S9) and β-catenin (S552 and S675) were greatly increased in the alpelisib-resistant cell lines (Figure 3H). Meanwhile, the subcellular localization of β-catenin in resistant cells was changed from the cell membrane to the cytoplasm and nucleus as demonstrated by nucleus/cytoplasm fractionation and immunofluorescence staining (Figure 3I-J).

It was reported that *ESR1* was the upstream transcription factor of *SGK3*. In our cell model, ESR1 was markedly upregulated in alpelisib-resistant breast cancer cells (Figure 3H). Knockdown of *ESR1* by siRNAs could significantly reduce the mRNA and protein levels of SGK3 as demonstrated by quantitative PCR and western blotting, confirming that *ESR1* was truly upstream of *SGK3* (Figure 3K-L). Taken together, enhanced stemness property and activated ESR1/SGK3/GSK3β/β-catenin pathway was discovered in the alpelisib-resistant breast cancer cells.

### SGK3 mediated the resistance to BYL719 and promoted breast cancer stemness phenotype

**Figure 4:**
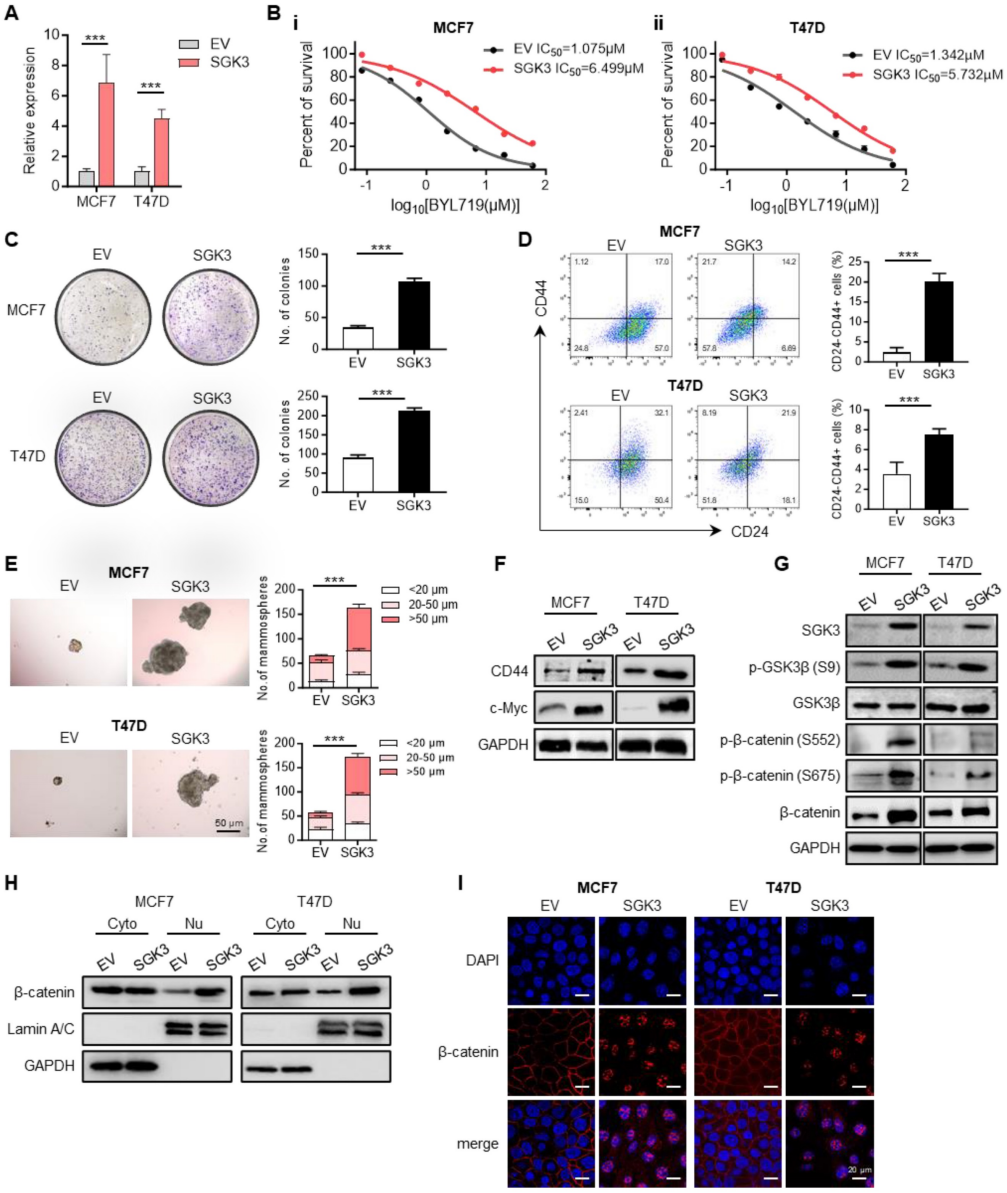
** SGK3 promoted breast cancer stemness and conferred resistance to BYL719 by activating β-catenin.** (A) Overexpression of SGK3 in MCF7 and T47D parental cells as shown by qPCR. (B) Dose-response curves for BYL719 in control and SGK3 overexpressed MCF7 and T47D cells. (C) Effect of SGK3 overexpression on colony formation. (D) The proportion of CD24-/CD44+ stem cell population in control and SGK3 overexpressed breast cancer cells. (E) Expression of CD44 and c-Myc in SGK3 overexpressed cells. (F) Effect of SGK3 overexpression on tumor sphere formation. (G) Western blot for total and phosphorylated GSK3β and β-catenin in SGK3 overexpressed cells. (H) Western blot showing the protein levels of β-catenin in cytoplasm and nucleus after SGK3 overexpression. (I) Immunofluorescence staining for β-catenin subcellular localization in control and SGK3 overexpressed MCF7 and T47D cells. Unpaired *t* test, ***p < 0.001.

To illustrate the role of SGK3 in alpelisib resistance, *SGK3* was ectopically expressed in parental MCF7 and T47D cells (Figure 4A). The overexpression of SGK3 in parental cells was sufficient to increase cell viability in presence of BYL719 as well as inavolisib (Figure 4B and Figure S3A-B). Colony formation assay showed that cells overexpressing SGK3 could generate more and larger clones than control cells (Figure 4C). The proportion of CD24-/CD44+ stem cell population was also significantly increased in cells overexpressing SGK3 (Figure 4D). Moreover, the tumor spheres formation ability was greatly increased in SGK3 overexpressed MCF7 and T47D cells (Figure 4E and Figure S3C-D). Concordant with functional experiments, SGK3 overexpression led to elevated expression of stem cell marker CD44 and c-Myc in breast cancer cells (Figure 4F). This set of experiments suggested that SGK3 was able to promote the stemness of breast cancer cells and resistance to BYL719. Next, the phosphorylation levels of GSK3β and β-catenin were examined to validate whether SGK3 could activate this pathway. Western blot results confirmed that overexpression of SGK3 significantly promoted the expression of p-GSK3β (S9) and β-catenin (S552 and S675) in breast cancer cells (Figure 4G). Phosphorated β-catenin resulted in the nuclear accumulation of β-catenin as confirmed by nucleus/cytoplasm fractionation and immunofluorescence in cells overexpressing SGK3 (Figure 4H-I). Thus, these results demonstrated that SGK3 could activated GSK3β/β-catenin signaling pathway and sustained the stemness of breast cancer cells.

### Knockdown of SGK3 sensitized resistant cells to BYL719 and enhanced the anti-tumor activity of alpelisib

**Figure 5:**
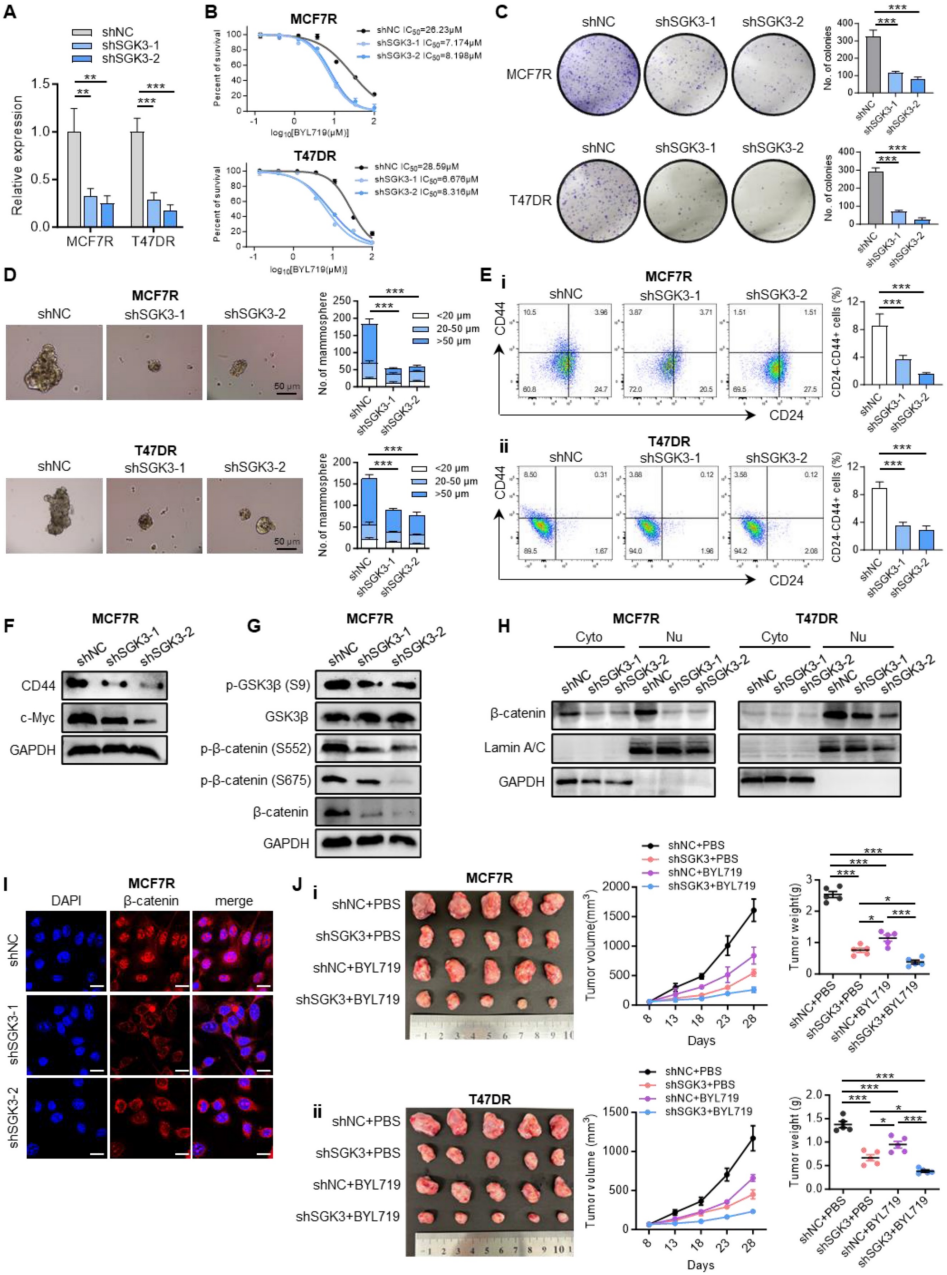
** Depletion of SGK3 restored the sensitivity to BYL719 and enhanced the anti-tumor activity of alpelisib.** (A) SGK3 knockdown efficiency in alpelisib-resistant cells MCF7R and T47DR. (B) Dose-response curves of BYL719 in control and SGK3 depleted MCF7R and T47DR cells. (C) Effect of SGK3 knockdown on colony formation. (D) Effect of SGK3 knockdown on tumor sphere formation. (E) The proportion of CD24-/CD44+ stem cell population after SGK3 knockdown as shown by flow cytometry. (F) Expression of stemness markers in SGK3-depleted cells. (G) Western blot showing the total and phosphorylated GSK3β and β-catenin after SGK3 knockdown. (H) Western blot showing the protein levels of β-catenin in cytoplasm and nucleus after SGK3 knockdown. (I) Immunofluorescence staining illustrating the subcellular translocation of β-catenin upon SGK3 knockdown. (J) Xenografts generated by orthotopically injection of control and SGK3-depleted MCF7R and T47DR cells with or without BYL719 treatment. In (A-E): **p < 0.01, ***p < 0.001 by unpaired *t* test. In (H): *p < 0.05, ***p < 0.001 by one-way ANOVA test.

To further validate the function of SGK3 in alpelisib resistance, MCF7R and T47DR cell lines stably expressing *SGK3* short hairpin RNAs (shRNA) were established ((Figure 5A). Upon SGK3 knockdown, the median lethal dose of BYL719 as well as inavolisib was markedly decreased in alpelisib-resistant cells, revealing that SGK3 knockdown re-sensitized resistant cells to PI3Kα inhibitors (Figure 5B and Figure S4A-B). Furthermore, SGK3 knockdown was significantly inhibited the colony formation and spheroid formation abilities of alpelisib-resistant MCF7R and T47DR cells (Figure 5C-D and Figure S4C-D). Meanwhile, the proportion of CD24-/CD44+ stem cell population was significantly lower in MCF7R and T47DR after SGK3 knockdown (Figure 5E). In line with decreased stemness phenotype, CD44 and c-Myc protein levels were significantly downregulated upon SGK3 knockdown (Figure 5F). Mechanically, knockdown of SGK3 significantly inhibited the expression of phosphorylated GSK3β (S9) and β-catenin (S552 and S675), which resulted in the degradation and subcellular translocation of β-catenin in alpelisib-resistant cells (Figure 5G-I). Therefore, these results demonstrated that SGK3 inhibition restored the sensitivity of resistant cells to BYL719 via suppression of stemness phenotype through GSK3β/β-catenin signaling pathway.

To investigate whether SGK3 could be a potential therapeutic target if resistance to alpelisib had been developed, MCF7R and T47DR cells with or without SGK3 manipulation were orthotopically injected into the mouse mammary fat pads and BYL719 treatment was given when the tumor volume reached about 100mm^3^. SGK3 knockdown resulted in a modest delay in tumor growth in shNC control xenografts (Figure 5J). Although some antitumor activity of BYL719 was observed in alpelisib-resistant cells, only the combination of BYL719 and SGK3 knockdown achieved the maximum inhibition of tumor growth (Figure 5J). Taken together, these results suggested SGK3 could be a promising target for alpelisib-resistant breast cancer.

### Combined suppression of SGK3 and PI3Kα in alpelisib-resistant cells

**Figure 6:**
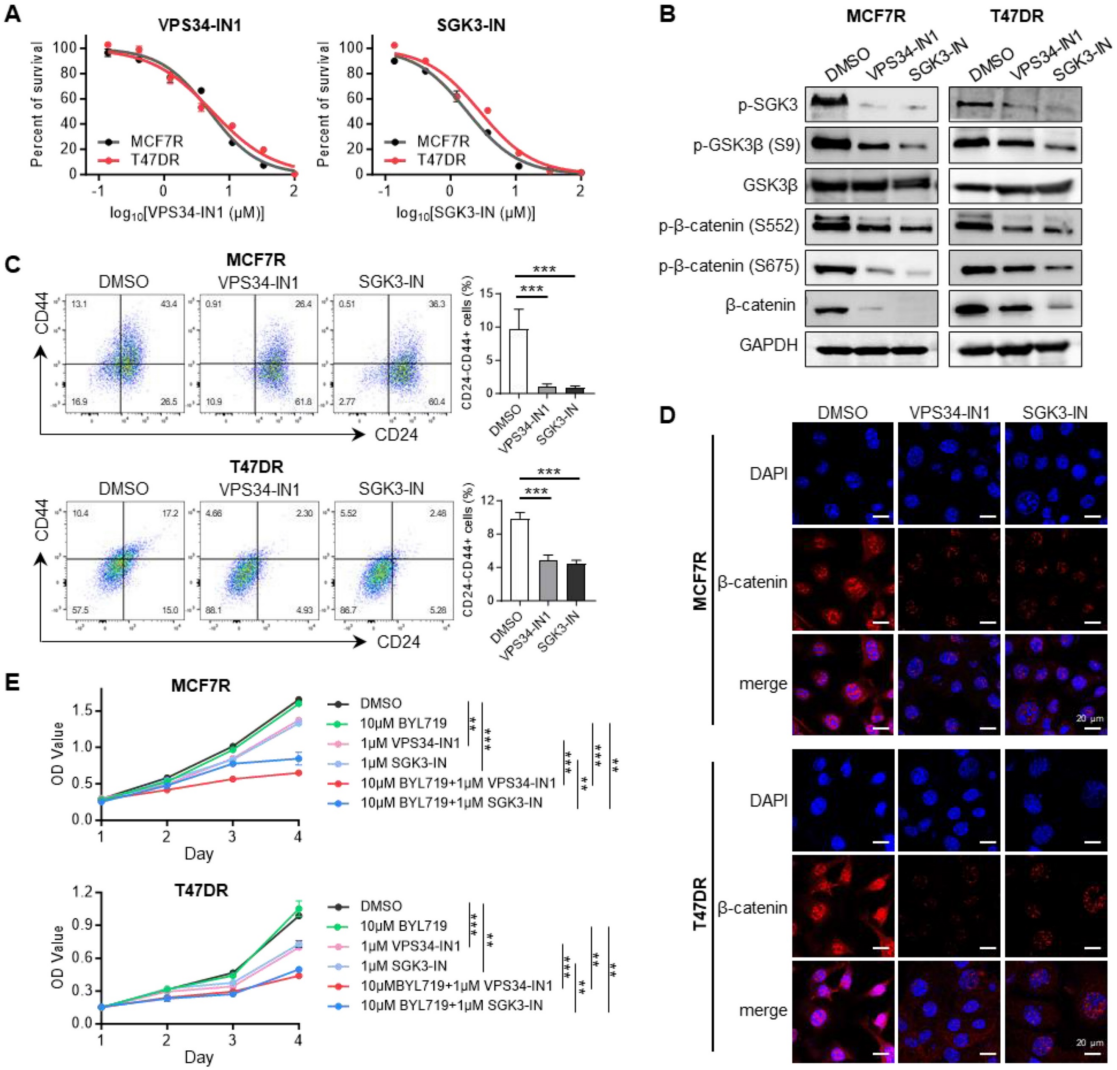
** SGK3 inhibition blocked β-catenin activation and reduced the stemness phenotype.** (A) Dose-response curves of SGK3 inhibitors namely VPS34-IN1 and SGK3-IN in alpelisib-resistant MCF7R and T47DR cells. (B) Western blot for total and phosphorylated SGK3, GSK3β and β-catenin after VPS34-IN1 and SGK3-IN treatment. (C) Effects of VPS34-IN1 and SGK3-IN on CD24-/CD44+ stem cell population in alpelisib-resistant cells. (D) Immunofluorescence images showing that the abundance and subcellular location of β-catenin in alpelisib-resistant cells treated with SGK3 inhibitors. (E) The growth curves of MCF7R and T47DR under the treatment of BYL719 and SGK3 inhibitors. Unpaired *t* test, **p < 0.01, ***p < 0.001.

To assess the effect of pharmacological inhibition of SGK3 in alpelisib-resistant cells, two reported SGK3 inhibitors, namely VPS34-IN1 and SGK3-PROTAC1 (SGK3-IN) were selected. The former drug is a potent and highly selective VPS34 inhibitor, which was reported to suppress SGK3 activation by reducing PtdIns(3)P levels 19, while the latter drug is a specific PROTAC conjugated SGK3 degrader, which could induce 50% SGK3 degradation within 2 hours 33. First, the inhibition of these two drugs on resistant cell growth was determined by CCK-8 assay (Figure 6A). As expected, the exposure of alpelisib-resistant cells to VPS34-IN1 and SGK3-IN significantly reduced the phosphorylation level of SGK3 protein, demonstrating the strong inhibition of these drugs on SGK3 activation (Figure 6B). Notably, the CD24-/CD44+ stem cell population was markedly decreased in alpelisib-resistant cells after VPS34-IN1 and SGK3-IN treatment, which was concordant with our previous results (Figure 6C). Western blot results also showed that the above two SGK inhibitors significantly decreased the expression of phosphorylated GSK3β and β-catenin and induced the degradation of β-catenin in nucleus (Figure 6B and 6D). Moreover, the maximum growth inhibition was observed in cells treated with combination of BYL719 and SGK3 inhibitors simultaneously, suggesting that SGK3 inhibition could enhance the antitumor activity of BYL719 (Figure 6E). Combinational index analysis further confirmed a synergistic interaction between SGK3-IN and BYL719 (Figure S5).

Taken together, based on our results, we propose a hypothesis that SGK3, a serum glucocorticoid kinase, is upregulated in alpelisib-resistant breast cancer cells and activates GSK3β/β-catenin signaling, which in turn promotes the stemness of breast cancer cells and ultimately promotes the resistance to alpelisib.

## Discussion

In this study, the PI3K/AKT signaling pathway in breast cancer cells was shown to be significantly inhibited by BYL719. By comparing the differences in biological characteristics between alpelisib-resistant cells and parental cells, we found that resistant cells had significantly enhanced proliferation ability and stemness *in vivo* and *in vitro*. Transcriptome profiles showed that *SGK3* was significantly highly expressed in the resistant cell lines. Both overexpression and knockdown experiments demonstrated that SGK3 was involved in the regulation of alpelisib resistance in breast cancer. Furthermore, we found that SGK3 was responsible for the phosphorylation and nuclear translocation of β-catenin by promoting the phosphorylation of GSK3β. More importantly, inhibitors of SGK3 including SGK3-IN and VPS34-IN1, could restored the sensitivity of drug-resistant cells to BYL719.

Although alpelisib (BYL719), a specific PI3K inhibitor, has been approved to treat advanced breast cancer patients 34, resistance to alpelisib is inevitable due to tumor evolution and selection. To investigate the molecular mechanisms involved in alpelisib resistance, the alpelisib-resistant breast cancer cell lines were successfully established. Both *in vivo* and *in vitro* experiments demonstrated the increased abilities of cell proliferation, migration and anti-apoptosis in alpelisib-resistant cells. Previous studies have suggested a population of cancer stem cells might be responsible for the resistance to targeted drug therapy 35, 36. These cells were featured by elevated CD44+/CD24- cell ratio, high aldehyde dehydrogenase activity and increased breast sphere formation ability 35, 36. However, whether cancer stem cells also contribute to alpelisib resistance in breast cancer remains unclear. In our study, the proportion of CD44+/CD24- stem cell population was significantly upregulated in alpelisib-resistant cells together with enhanced expression levels of important stemness markers. Based on the above results, we speculated that there were some changes in certain genes or signaling pathways in these alpelisib-resistant cells that could induce cancer stemness, further leading to drug resistance.

SGK3, a serine/threonine-protein kinase, has been implicated in acquired resistance to multiple drugs, such as aromatase inhibitors and rapamycin 19, 23, 24. However, whether SGK3 also plays an important role in alpelisib-related resistance is scarcely investigated. In our model, *SGK3* and *ESR1* were markedly upregulated in alpelisib-resistant breast cancer cell lines. Knockdown of ESR1 in resistant cell lines significantly reduced SGK3 expression, consistent with previous studies indicating SGK3 was a transcriptional target of the estrogen receptor 37. It was reported that long-term inhibition of class I PI3K could promote cancer stem cell expansion by phosphorating GSK3β and consequently increasing the stability of β-catenin 25, 38. In our study, SGK3 was demonstrated to be responsible for the resistance to BYL719 and enhanced breast cancer stemness by increasing the phosphorylation levels of GSK3β and β-catenin. This phosphorylation subsequently led to the translocation of β-catenin from the cell membrane and cytoplasm to the nucleus, where it became activated.

Inavolisib is another FDA approved PI3K inhibitors, showing promising antitumor activity in advanced, *PI3KCA*-mutated, HR+ breast cancer 39, 40. As expected, alpelisib-resistant cells also acquired resistance to inavolisib. Interesting, we also confirmed that knockdown of SGK3 could re-sensitize resistant cells to both BYL719 and inavolisib, enhancing the anti-tumor effect of alpelisib *in vivo*. To investigate common mechanisms involving in drug-resistance to all PI3K inhibitors, additional PI3K inhibitor-resistant cells should be developed separately by treating them with individual PI3K inhibitor.

SGK has been proposed as a promising target for cancer therapy, hence SGK inhibitors have been developed and tested based on this purpose 41. Notably, VPS34-IN1 has been shown to inhibit the phosphorylation of SGK3, while SGK3-PROTAC1 is able to suppress both SGK3 expression and phosphorylation 17. In our study, both VPS34-IN1 and SGK3-PROTAC1 exhibited cytotoxic effects on the alpelisib-resistant breast cancer cells. We also demonstrated that these two SGK3 inhibitors could repress the stemness phenotype and restore the sensitivity of the resistant cell lines to BYL719. Moreover, a synergistic interaction between SGK3-PROTAC1 and BYL719 was demonstrated by CCK-8 assay, suggesting that SGK3 inhibitors should be promising treatment strategies for breast cancer patients who had developed resistance to alpelisib. Unfortunately, we did not exam the therapeutic effects of SGK3 inhibitors on alpelisib resistance by *in vivo* model.

In summary, our findings showed that SGK3 mediated resistance to PI3Kα inhibitors through the activation of GSK3β/β-catenin signaling pathway and highlighted the therapeutic potential of the combination treatment of SGK3 inhibitors and alpelisib for patients developing resistance to both endocrine-based treatments and PIK3CA inhibitors.

## Supplementary Material

Supplementary figures and tables.
