# Supplementary figures and images for: The SGK3/GSK3β/β-catenin signaling promotes breast cancer stemness and confers resistance to alpelisib therapy
Source: Int J Biol Sci. 2025 Mar 19;21(6):2462–75. doi: 10.7150/ijbs.104850 (PMC12035905; doi:10.7150/ijbs.104850)

Figure S1

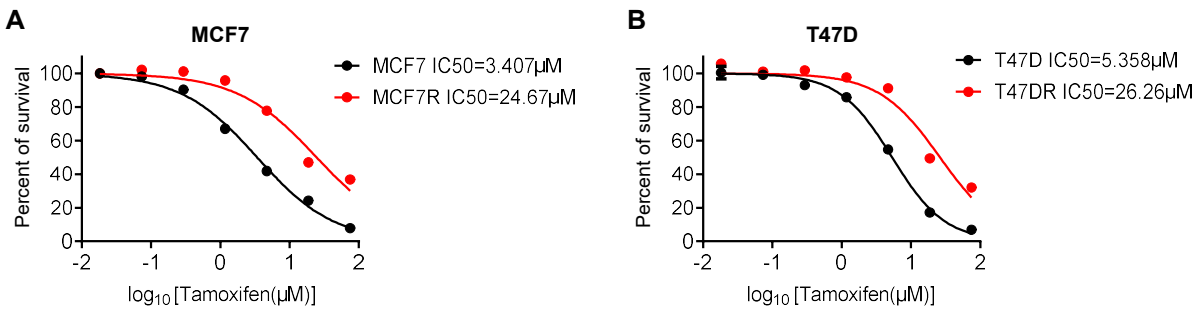

**Figure S2**

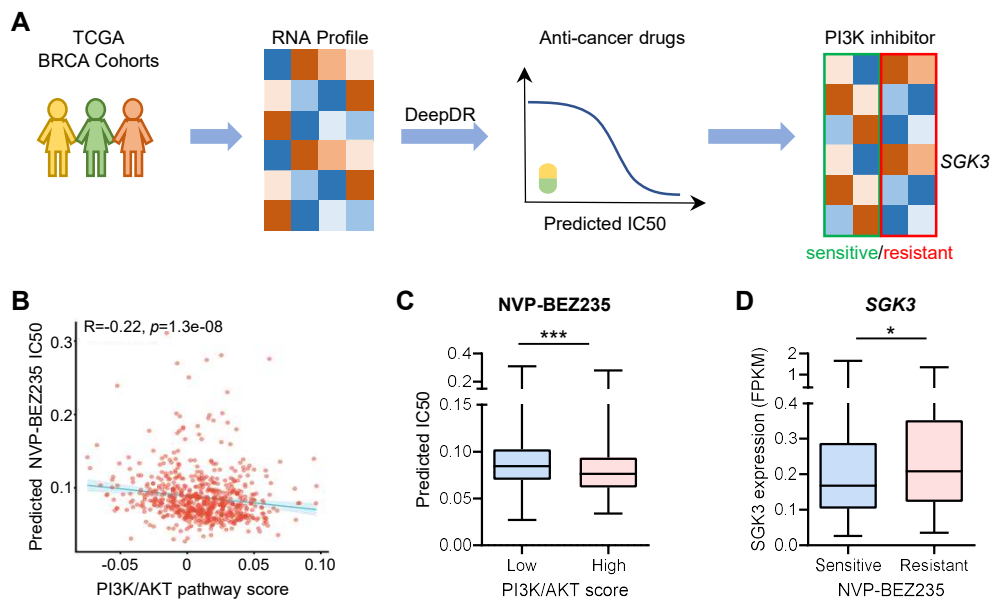

Figure S3

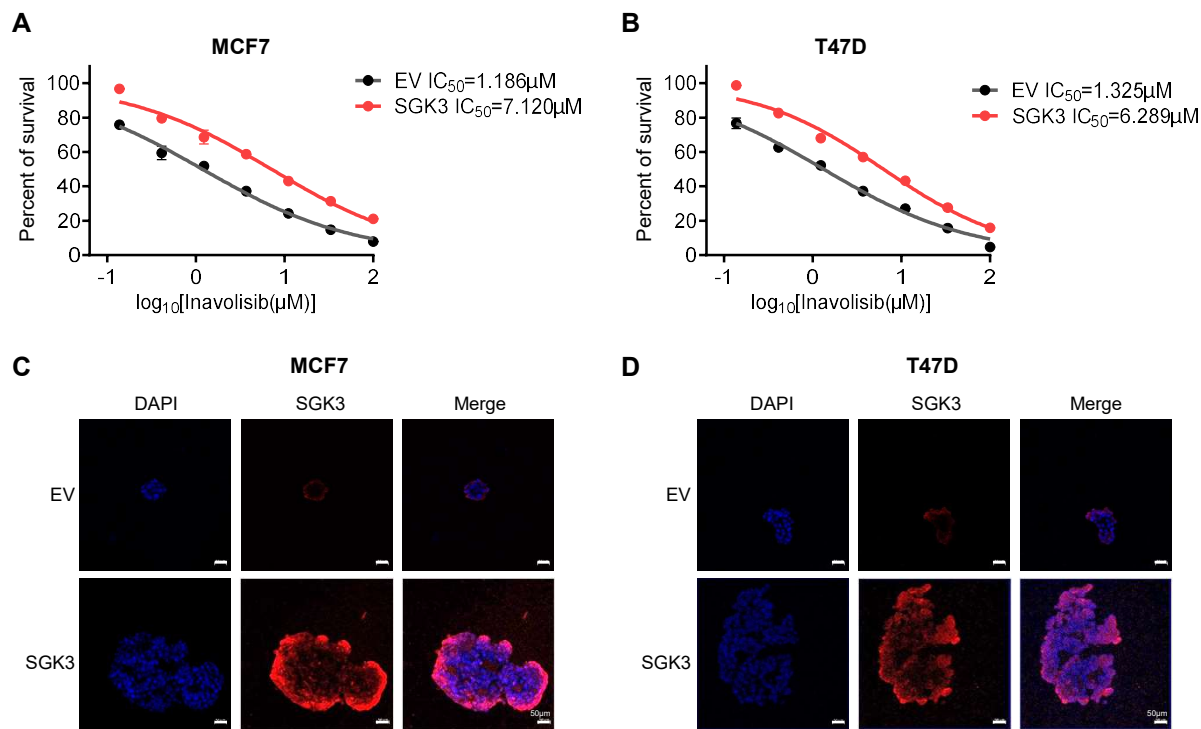

Figure S4

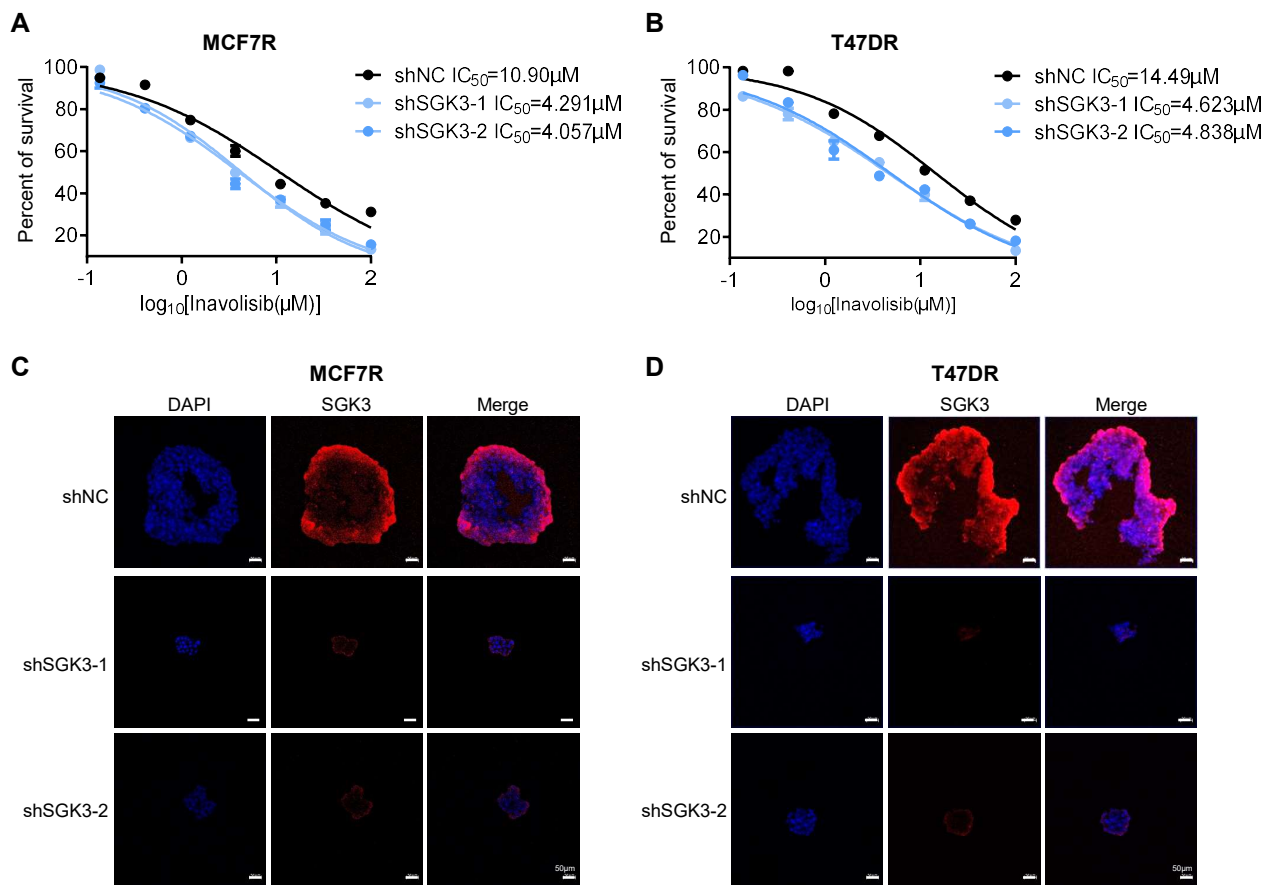

Figure S5

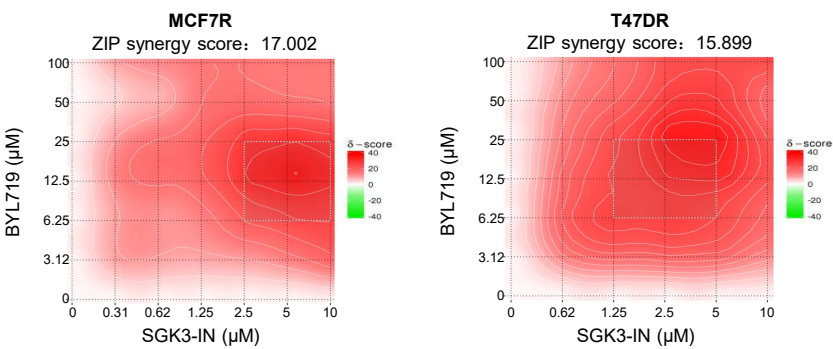

Supplement: Supplementary file 1 — Supplementary figures and tables. [file ijbsv21p2462s1.zip › supp Figures.pdf]
